# Histogram of Oriented Gradient Based Gist Feature for Building Recognition

**DOI:** 10.1155/2016/6749325

**Published:** 2016-10-31

**Authors:** Bin Li, Kaili Cheng, Zhezhou Yu

**Affiliations:** ^1^School of Information Engineering, Northeast Electric Power University, Jilin 132012, China; ^2^School of Computer Science and Technology, Jilin University, Changchun 130012, China

## Abstract

We proposed a new method of gist feature extraction for building recognition and named the feature extracted by this method as the histogram of oriented gradient based gist (HOG-gist). The proposed method individually computes the normalized histograms of multiorientation gradients for the same image with four different scales. The traditional approach uses the Gabor filters with four angles and four different scales to extract orientation gist feature vectors from an image. Our method, in contrast, uses the normalized histogram of oriented gradient as orientation gist feature vectors of the same image. These HOG-based orientation gist vectors, combined with intensity and color gist feature vectors, are the proposed HOG-gist vectors. In general, the HOG-gist contains four multiorientation histograms (four orientation gist feature vectors), and its texture description ability is stronger than that of the traditional gist using Gabor filters with four angles. Experimental results using Sheffield Buildings Database verify the feasibility and effectiveness of the proposed HOG-gist.

## 1. Introduction

Building recognition is becoming increasingly more interesting to researchers, since it can be applied to many real-world problems, such as robot vision or localization [1], mobile device navigation [2, 3], and building labeling in videos. However, building recognition is a challenging task because building images could be taken from different viewpoints under different lighting conditions or suffering from occlusion from billboard, trees, vehicles, or other buildings. The biggest difficulty for build recognition is to design a feature extraction algorithm that can accurately and completely describe building characteristics.

Interest points extracted by the Harris corner detector were applied to matching buildings in the world space for mobile device [3]. Li and Shapiro [4] used the consistent line cluster for content-based image retrieval. Specifically, the color, orientation, and spatial features of line segments are exploited to group image into line clusters. The intracluster and intercluster relationships were used to recognize buildings in photographic images. Zhang and Košecká [5] proposed a hierarchical building recognition method that has two steps and bases on the localized color histograms. The first step uses localized color histograms, and in the second step the method refined matching SIFT descriptors. Fritz et al. [6] applied the “Informative Descriptor Approach” on SIFT features (i-SIFT descriptors) and proposed a robust building detection and recognition method.

In [7], Li and Allinson pointed out the following: all the mentioned building recognition algorithms have two drawbacks. (1) They are based on the detection of low-level features such as vanishing points and line segments. The representation of building characteristics is restricted, since these low-level features cannot reveal the truly semantic concepts of building images. (2) If these raw high-dimensional feature vectors were used for recognition which may cause large memory requirements, it would result in high computational cost. Li and Allinson proposed a new building recognition method to address these two drawbacks. Li and Allinson use the gist feature extraction approach proposed by Siagian and Itti to obtain gist features of building images. In Siagian and Itti's gist extraction method [8], 34 feature maps are created by filtering of the original image in orientation channels, color channels, and intensity channels in multiple spatial scales. Each feature map is divided into a 4 × 4 grid, and the mean values of each grid were calculated to produce 16 values for a gist vector. As a result, the original image is represented by a 544-dimension feature vector. To reduce computational costs and preserve discriminative information as much as possible, several manifold learning dimensionality reduction algorithms, such as principal component analysis (PCA) [9], locality preserving projections (LPP) [10], and linear discriminant analysis (LDA) [11], are used for dimensionality reduction before recognition. The gist feature extraction and dimensionality reduction-based building recognition method proposed by Li and Allinson has been proven to be more effective than those low-level feature methods [3–7]. Li et al. [12] proposed subregion's multiscale gist feature (SM-gist) extraction method. The SM-gist divided building image into 4 × 5 subregions, and gist vectors are extracted from these subregions individually. The interference of nonuniform illumination is mitigated by the SM-gist extraction method. Zhao et al. [13] proposed multiscale gist (MS-gist) feature for building recognition. The MS-gist features can be stable to capture the representation features of the building images with rotation, variant lighting conditions, and occlusions.

The gist feature extraction methods proposed by Siagian and Itti were originally used for the task of scene recognition, but building recognition is different from scene recognition. This is because there are many lines on the building surface. For building recognition tasks, the texture feature of buildings is more important than the color and intensity features. Siagian and Itti's gist feature extraction method used Gabor filters with only four angles to extract the orientation information. So, the texture description ability of Siagian and Itti's gist feature extraction method is not good. To improve the texture description ability of Siagian and Itti's gist feature extraction method, we propose histogram of oriented gradient based gist (HOG-gist) feature extraction method. The histogram of oriented gradient (HOG) was first proposed by Dalal and Triggs [14]. Due to the strong texture and shape description ability, the HOG can be used in human detection [14], face recognition [15, 16], image registration [17], and many other tasks [18–21]. Our proposed HOG-gist extraction method individually computes the normalized histograms of multiorientation gradients for the same image with four different scales. These normalized histograms of oriented gradients are orientation gist feature vectors of an image. These orientation gist vectors combined with intensity and color gist feature vectors proposed by the traditional method are the proposed HOG-gist vectors.

This paper is organized as follows: we give a briefly review of Siagian and Itti's gist feature extraction method in Section 2; the histogram of oriented gradient based gist (HOG-gist) feature extraction method is proposed in Section 3; recognition performance on the Sheffield Buildings Database is detailed in Section 4, while Section 5 concludes the paper.

## 2. Gist Feature Extraction

In this section, we give a brief review of Siagian and Itti's gist feature extraction method [8] and the building recognition method proposed by Li and Allinson [7].

The psychological research [22] has proven that human can grasp the “gist” of an image by glancing at it for just a few seconds. Siagian and Itti's gist feature extraction method is aiming to simulate this ability of human beings. Siagian and Itti's gist feature extraction method has two main steps: saliency feature map construction and gist feature extraction. Saliency feature maps are constructed based on low-level visual features, including the intensity channel, color channel, and orientation channel, which are extracted in parallel. Equation (1) is utilized to compute the intensity channel [8, 23]:

(1) $$\begin{array}{c} I=\left(\;r+g+b\;\right). \end{array}$$

*R*, *G*, *B*, *Y* color channel [8, 23] can be obtained by the following equations:

(2) R=r−g+b2,G=g−r+b2,B=b−r+g2,Y=r+g2−r−g2−b,

where *r*, *g*, *b* represent the red, green, and blue channels of the RGB color space of the original image.

For the intensity channel and the color channel, five image Gaussian pyramids, *I*(*σ*), *R*(*σ*), *G*(*σ*), *B*(*σ*), and *Y*(*σ*), with nine spatial scales, ranging from 1 : 1 (scale zero) to 1 : 256 (scale eight) in eight octaves, are created [8, 23], where *σ* = 0,1,…, 8. The intensity and color saliency feature maps can be obtained by applying the center-surround operation to these Gaussian pyramids. The center-surround operation defined by Siagian and Itti is as follows [8, 23]: a pixel at scale *c* = {2,3, 4} is the center, and the corresponding pixels at scale *s* = *c* + *δ*, where *δ* = {3,4} is the surround. From (3), we can get six intensity feature maps [8, 23], and twelve color feature maps [8, 23] are obtained by (4):

(3) Ic,s=Ic⊝Is,

(4) RGc,s=Rc−Gc⊝Gs−Rs,

where ⊝ denotes the cross-scale difference between two images in a Gaussian pyramid.

Gabor filters with four different scales *c* = {1,2, 3,4} and four orientations *θ* = {0°, 45°, 90°, 135°} are applied to the intensity channel *I* and extract the 16 orientation feature maps [8, 23].

In total, 34 saliency feature maps are computed: 6 for intensity, 12 for color, and 16 for orientation.

**Figure 1:**
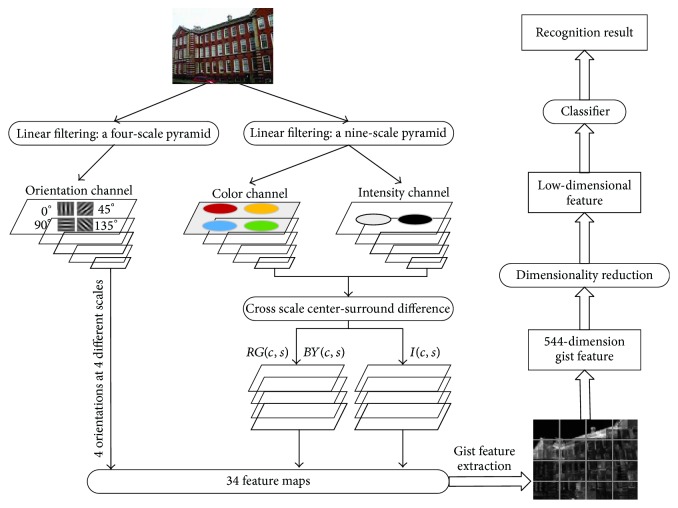
The building recognition method proposed by Li and Allinson.

Each map is then divided into 4 × 4 grid subregions, and then take the mean of each grid to produce 16 values for the 16-dimension gist feature vector. We can get 34 gist feature vectors from the 34 feature maps. The 34 gist feature vectors included 6 intensity gist feature vectors, 12 color gist feature vectors, and 16 orientation gist feature vectors. The combination of all the gist feature vectors is a 544-dimension feature vector. Therefore, each building image can be represented by this 544-dimension feature vector.  Figure 1 shows the main progress of by Li and Allinson's building recognition method [7]. In Figure 1, Siagian and Itti's gist feature extraction method is used to extract the gist features from building images. Then, dimensionality reduction algorithm is used to reduce the dimension of the original feature vectors from 544 to a much lower dimension before classification.

## 3. Histogram of Oriented Gradient Based Gist Feature (HOG-gist) Extraction

In this section, we will introduce in detail our histogram of oriented gradient based gist feature (HOG-gist) extraction method and our building recognition method.

### 3.1. Orientation Gist Feature Extraction

**Figure 2:**
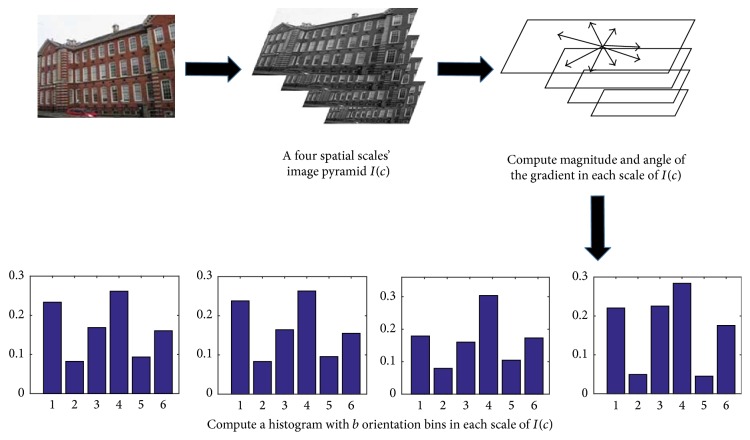
The process of the orientation gist feature extraction.

The orientation gist features can be extracted by the following five steps. This process is shown by Figure 2.

The process is as follows:

(1) An image pyramid *I*(*c*){*c* = 0,1, 2,3} is created on the intensity channel *I* (see (1)) with four spatial scales ranging from 1 : 1 (scale zero) to 1 : 8 (scale three) in four octaves. A histogram of oriented gradient will be computed in each scale of *I*(*c*).
(2) Use gradient filter [−1,0, 1] with no smoothing [14, 15] to compute the horizontal *G*_*x*_(*x*, *y*) and vertical *G*_*y*_(*x*, *y*) gradient of *I*(*c*).
(3) Compute magnitude |*G*(*x*, *y*)| and angle *θ*(*x*, *y*) of the gradient [14, 15]:
  (5) Gx,y=Gxx,y2+Gyx,y2,θx,y=arctan⁡Gyx,yGxx,y.
(4) Compute a histogram with *b* orientation bins in 0°–180°. Magnitude (|*G*(*x*, *y*)|) whose angle *θ*(*x*, *y*) belongs to the same bin will be added up as the value of this bin. The value of *b* is determined according to experimental results in Section 4.1.
(5) The histograms can be normalized by *L*2 − *Hys* (Lowe-style clipped L2 norm) [14] normalization method.

After the computation of all the histograms of oriented gradient in four scales, we can get four *b*-dimension vectors which are the orientation gist feature vectors of HOG-gist; namely, the orientation gist feature vectors of the HOG-gist are these four histograms of oriented gradient.

### 3.2. Our Proposed Building Recognition Method

**Figure 3:**
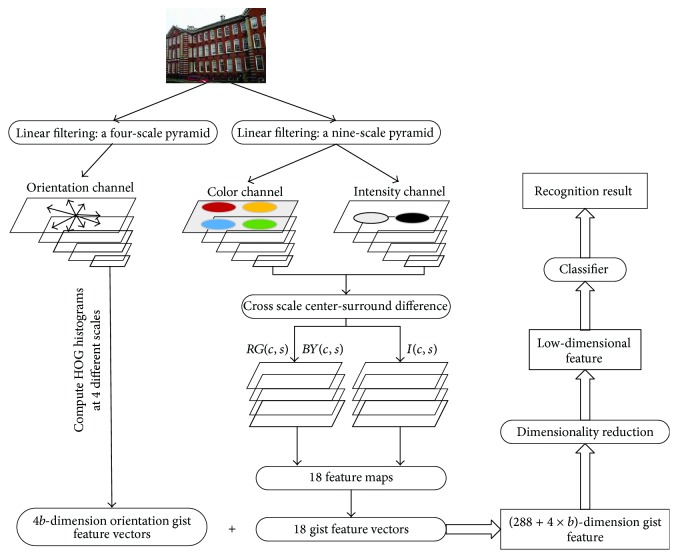
The building recognition method based on HOG-gist.

Figure 3 shows the building recognition method based on our HOG-gist. The orientation channel in Figure 3 refers to the extraction procession of orientation gist feature, which has been explained in detail in Figure 2 and Section 3.1.

In Figure 3, the color channel and intensity channel present the procession of extraction in color gist feature vector and intensity gist feature vector of the building Image. The above extraction methods of gist feature vector are as same as the traditional method shown in Figure 1. After the procession of the color channel and intensity channel, 6 intensity gist feature vectors and 12 color gist feature vectors have been obtained from the intensity channel and the color channel, respectively. Then, 6 intensity gist feature vectors, 12 color gist feature vectors, and 4 orientation gist feature vectors will be combined to our finally proposed HOG-gist. Each intensity gist feature vector and color gist feature vector are of 16-dimension vector. In addition, orientation gist feature vector is *b* dimension. Therefore, HOG-gist equals a (288 + 4 × *b*)-dimension (6 × 16 + 12 × 16 + 4 × *b* = 288 + 4 × *b*) gist feature vector. The HOG-gist will reflect the characteristics of the original building image.

Then, dimensionality reduction algorithms (such as LPP [10], MFA [24], PCA [9], and NPE [25]) have been applied to HOG-gist feature vector for the feature vector with lower dimension. Finally, the feature vector of lower dimension will be classified via the classifiers, such as Nearest Neighbor Classifier (NN) [26], Support Vector Machine (SVM) [27], and BP-neural Network (BP) [28].

## 4. Experiments

**Figure 4:**
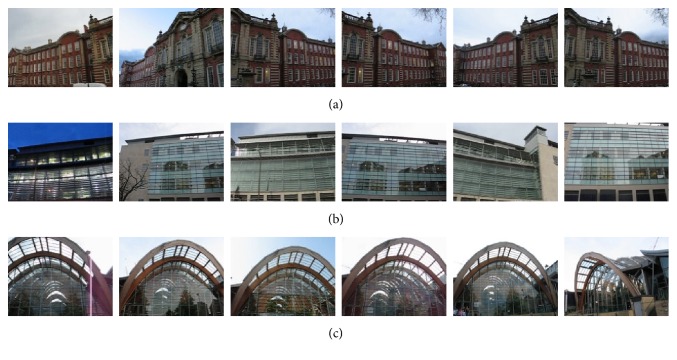
(a)–(c) are sample images from categories 1, 10, and 31, respectively.

To evaluate the performance of HOG-gist, we carry out experiments on the Sheffield Buildings Database [29]. The Sheffield Buildings Database contains 3192 building images of 40 buildings, and for each building the number of building images varies from 100 to 400. The size of these images is 160 × 120.  Figure 4 shows sample images of the Sheffield Buildings Database. From Figure 4, we can see that buildings are taken from different viewpoints and images may be under different scaling and illumination conditions, and there are occlusion and rotation phenomena in some of the images.

The number of building images of each building is different, so we select the first 20 images from each building and form a subset which we name as D1. D1 consists of 40 buildings and 20 images for each building. So, D1 consists of 800 buildings in total.

In our experiments, D1 was partitioned into different sample collections. We let *G*_*m*_/*P*_*n*_ indicate that, for each building in D1, *m* images were selected at random for training and the remaining *n*  (*n* = 20 − *m*) images were employed for testing. For each *G*_*m*_/*P*_*n*_, 50 random splits are generated and the final result of this *G*_*m*_/*P*_*n*_ is obtained by taking the mean of the 50 recognition accuracy values.

### 4.1. Experiments for Parameter Selection

**Figure 5:**
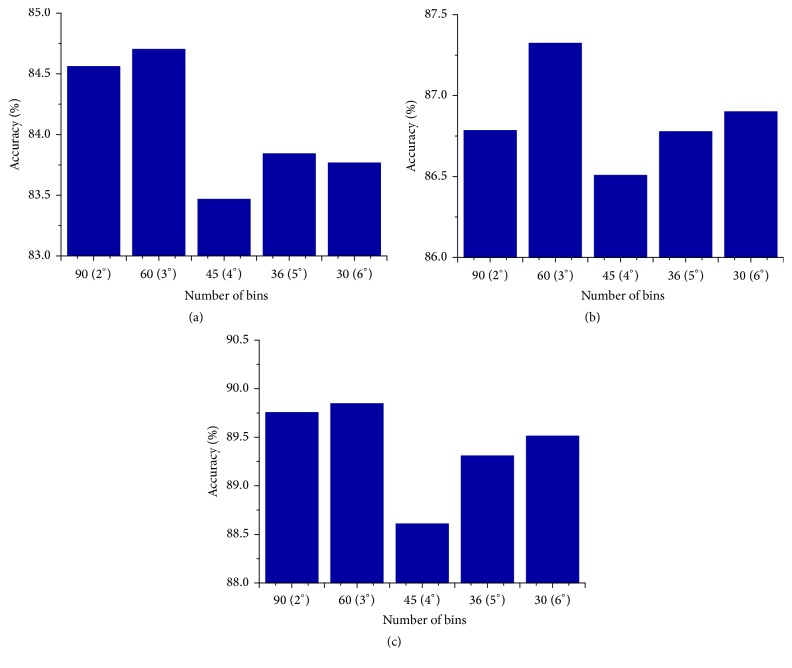
The mean recognition accuracy values of each value of parameter *b*. (a) *G*_4_/*P*_16_ of D1. (b) *G*_5_/*P*_15_ of D1. (c) *G*_6_/*P*_14_ of D1.

In this subsection, we aim at choosing a proper parameter, *b*, which is the number of orientation bins of histogram of oriented gradient for our HOG-gist. We compute a histogram with *b* orientation bins in the interval (0°–180°). If the step length of an angle is 2°, 3°, 4°, 5°, and 6°, *b* will be 90 (180°/2°), 60 (180°/3°), 45 (180°/4°), 36 (180°/5°), and 30 (180°/6°), respectively. Parameter selection experiments are conducted on *G*_4_/*P*_16_, *G*_5_/*P*_15_, and *G*_6_/*P*_14_ of the D1 subset, respectively (Figure 5). In this experiment, LPP [10] was used for dimensionality reduction; at the same time, classification is conducted based on the Nearest Neighbor Classifier (NN) [26]. The mean recognition rate corresponding to each value of *b* is shown in Figures 4(a)–4(c).

From Figures 4(a)–4(c), it can be seen that HOG-gist achieves the highest recognition rate when the value of *b* is 60. As a result, we set the value of *b* to 60 in the following experiments. Since there are 60 bins values in the histogram, the dimension of an orientation gist feature vector is 60. There are four 60-dimension orientation gist feature vectors extracted by the HOG-gist extraction method. The HOG-gist is a 528-dimensional gist feature vector, whose dimension is similar to the dimension of Siagian and Itti's gist.

### 4.2. Building Recognition Using Different Dimensionality Reduction Algorithms

In this experiment, we evaluated the performance of our HOG-gist by comparing HOG-gist with Siagian and Itti's gist. LPP [10], NPE [25], PCA [9], and MFA [24] are employed as the dimensionality reduction algorithm, respectively. Finally, classification is conducted based on the Nearest Neighbor Classifier (NN) [26]. The mean accuracy values of Siagian and Itti's gist and our HOG-gist are listed in line 1 and line 2 of each Table, respectively.

From the results shown in Tables 1 [Table tab2] [Table tab3]–4, one can find the following:

1. With the increasing number of training samples, the mean recognition rates of the two gist feature extraction methods have risen differently.
2. Our HOG-gist shows a better performance than Siagian and Itti's gist regardless of which kind of dimension reduction algorithm is selected.
3. In most situations, feature dimensions of our HOG-gist corresponding to the best recognition results are much lower than those of Siagian and Itti's gist. This indicates that the texture and shape description ability of our HOG-gist are better than those of Siagian and Itti's gist. Therefore, our HOG-gist feature can be reduced to a lower dimension. Then the higher recognition rate of HOG-gist is achieved.
4. MFA is a supervised subspace learning dimension reduction algorithm. The average recognition rate of HOG-gist combined with MFA is higher than that of HOG-gist combined with other dimension reduction algorithms, which is the same to Siagian and Itti's gist feature.
5. As an unsupervised dimension reduction algorithm, the performance of NPE is satisfied. The mean recognition accuracy values of HOG-gist combined with NPE are only slightly lower than those of HOG-gist combined with MFA.

### 4.3. Building Recognition Using Different Classifiers

**Figure 6:**
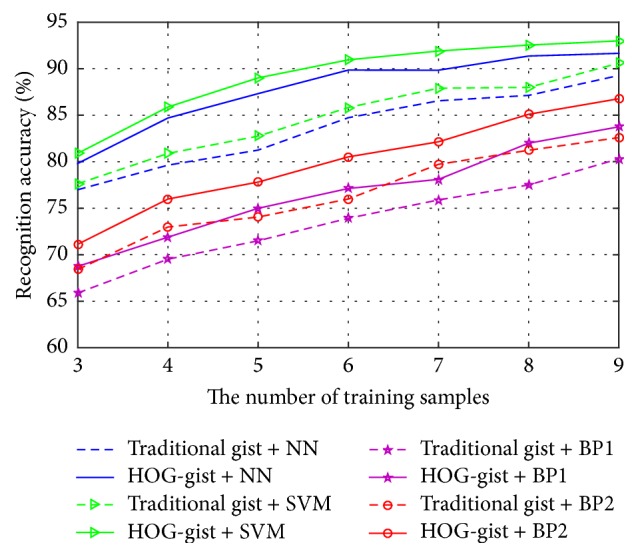
The mean recognition accuracy of HOG-gist or traditional gist (Siagian and Itti's gist) combined with different classifiers on the Sheffield Buildings Database.

Building recognition was conducted by combining HOG-gist or traditional gist (Siagian and Itti's gist) with different classifiers to compare the performances of HOG-gist and traditional gist in this experiment. LPP algorithm is the dimensionality reduction algorithm of HOG-gist. And then, the low-dimensional features after dimensionality reduction were classified individually by using four different classifiers: Nearest Neighbor Classifier (NN), SVM with the radial base kernel function, and BP-neural Network with two and three hidden layers. The two BP-neural Networks are denoted as BP1 and BP2 in Figure 6, respectively. Then, the above experiments were repeated for Siagian and Itti's gist feature.

The mean recognition results are in Figure 6. In Figure 6, the solid line shows the result of HOG-gist combined with a certain classifier, and the dashed line in the same color is the result of Siagian and Itti's gist combined with the same classifier. The horizontal axis of Figure 6 is the number of training samples, and the vertical axis represents the mean recognition accuracy corresponding to each number of training samples.

From Figure 4, we can make the following conclusions:

1. No matter which classifier combined with the HOG-gist, it has gained higher mean recognition rate than the traditional gist (Siagian and Itti's gist) combined with the same classifier, which shows that the HOG-gist is superior to Siagian and Itti's gist feature.
2. With SVM, the HOG-gist has achieved the highest mean recognition rate; the second highest recognition rate is with NN, and the lowest recognition rate is with BP. Siagian and Itti's gist feature combined with the above classifier also got the same result.
3. The mean recognition rate of Siagian and Itti's gist with SVM is higher than the recognition rate of HOG-gist with BP or NN, which shows that the selection of classifier is as important as the selection of feature extraction method.

## 5. Conclusions

There are a lot of lines on the building surface, so the texture feature of buildings is more important than the color feature and intensity feature for building recognition tasks. In order to improve the texture description ability of traditional gist feature extraction method, we proposed histogram of oriented gradient based gist (HOG-gist) feature extraction method. Our method employs the normalized histograms of oriented gradients as orientation gist feature vectors of an image. These orientation gist vectors combined with intensity and color gist feature vectors extracted by the traditional method are our HOG-gist. The HOG-gist contains four multiorientation histograms (four orientation gist feature vectors), and its texture description ability is stronger than that of the traditional gist using Gabor filters with four angles. The HOG-gist is a 528-dimensional gist feature vector, whose dimension is similar to the dimension of Siagian and Itti's gist, but its mean recognition accuracy is better than the mean recognition accuracy of Siagian and Itti's gist.

## Figures and Tables

**Table 1:** Mean recognition accuracy of Siagian & Itti's gist + LPP and HOG-gist + LPP. The numbers in parentheses indicate the corresponding feature dimensions which give the best results after dimensionality reduction.

	*G*_3_/*P*_17_	*G*_4_/*P*_16_	*G*_5_/*P*_15_	*G*_6_/*P*_14_	*G*_7_/*P*_13_	*G*_8_/*P*_12_	*G*_9_/*P*_11_
Siagian & Itti's gist + LPP	76.99 (70)	79.63 (80)	81.25 (85)	84.73 (90)	86.55 (95)	87.13 (97)	89.27 (98)
SM-gist + LPP	79.82 (65)	84.70 (69)	87.32 (79)	89.85 (64)	89.84 (36)	91.36 (57)	91.65 (63)

**Table 2 tab2:** Mean recognition accuracy of Siagian & Itti's gist + NPE and HOG-gist + NPE. The numbers in parentheses indicate the corresponding feature dimensions which give the best results after dimensionality reduction.

	*G*_3_/*P*_17_	*G*_4_/*P*_16_	*G*_5_/*P*_15_	*G*_6_/*P*_14_	*G*_7_/*P*_13_	*G*_8_/*P*_12_	*G*_9_/*P*_11_
Siagian & Itti's gist + NPE	77.80 (58)	80.53 (67)	82.15 (75)	85.98 (70)	87.05 (85)	88.95 (94)	90.98 (96)
SM-gist + NPE	81.81 (50)	85.76 (54)	87.68 (59)	90.52 (61)	91.67 (63)	91.76 (67)	92.71 (70)

**Table 3 tab3:** Mean recognition accuracy of Siagian & Itti's gist + PCA and HOG-gist + PCA. The numbers in parentheses indicate the corresponding feature dimensions which give the best results after dimensionality reduction.

	*G*_3_/*P*_17_	*G*_4_/*P*_16_	*G*_5_/*P*_15_	*G*_6_/*P*_14_	*G*_7_/*P*_13_	*G*_8_/*P*_12_	*G*_9_/*P*_11_
Siagian & Itti's gist + PCA	81.82 (54)	82.33 (46)	83.10 (35)	84.62 (54)	85.25 (71)	87.40 (77)	90.17 (73)
SM-gist + PCA	81.96 (42)	85.34 (29)	87.48 (31)	89.30 (62)	90.03 (66)	91.82 (69)	92.28 (72)

**Table 4:** Mean recognition accuracy of Siagian & Itti's gist + MFA and HOG-gist + MFA. The numbers in parentheses indicate the corresponding feature dimensions which give the best results after dimensionality reduction.

	*G*_3_/*P*_17_	*G*_4_/*P*_16_	*G*_5_/*P*_15_	*G*_6_/*P*_14_	*G*_7_/*P*_13_	*G*_8_/*P*_12_	*G*_9_/*P*_11_
Siagian & Itti's gist + MFA	80.52 (47)	84.50 (47)	86.84 (62)	88.83 (63)	90.30 (76)	91.19 (78)	92.20 (79)
SM-gist + MFA	82.41 (48)	86.29 (46)	88.72 (41)	91.25 (58)	92.83 (64)	93.65 (37)	94.41 (66)
